# Revolutionizing Mushroom processing: Innovative techniques and technologies

**DOI:** 10.1016/j.fochx.2024.101774

**Published:** 2024-08-25

**Authors:** Sangeeta, Dhriti Sharma, Seema Ramniwas, Robert Mugabi, Jalal Uddin, Gulzar Ahmad Nayik

**Affiliations:** a Department of Agriculture & Food Processing, Guru Nanak College, Budhlada, Mansa, Punjab, India; b University Centre for Research and Development, Chandigarh University, Gharuan, Mohali 140413, Punjab, India; c Department of Food Technology and Nutrition, Makerere University, Kampala, Uganda; d Department of Pharmaceutical Chemistry, College of Pharmacy, King Khalid University, Abha 61421, Saudi Arabia.; e Marwadi University Research Centre, Department of Microbiology, Marwadi University, Rajkot, Gujarat 360003, India

**Keywords:** Mushroom, Innovative processing, High-pressure processing, Nuclear magnetic resonance, Automation

## Abstract

In recent years, the global mushroom industry has seen remarkable growth due to its nutritional benefits, increasing market value, and rising consumer demand. Mushrooms are valued for their unique flavor, low sugar and salt, and rich Vitamin D content. In India as well as across the globe, mushroom cultivation is becoming increasingly popular among new entrepreneurs, leveraging the diverse agro-climatic conditions and substantial agricultural waste. Various government policies are also fostering research and development in this sector. To extend shelf life and preserve quality, various preservation techniques are employed, including drying, freezing, canning, high-pressure processing and modified atmosphere packaging. Furthermore, cutting-edge technologies such as nuclear magnetic resonance and spectroscopy are improving post-harvest processing, helping to maintain sensory properties and nutritional content. Automation is also transforming mushroom processing by enhancing efficiency and scalability. This review examines the innovative methods and technologies driving advancements in mushroom production and quality worldwide.

## Highlights

• Mushroom cultivation offers a profitable agribusiness, enhancing resource use, circular economy, and nutrition.
• Mushrooms are increasingly used in soups, meats, and other processed foods, supported by automation.
• NMR, imaging, and spectroscopy maintain sensory & nutritional properties of mushroom.
• Advanced methods like HPP and MAP enhance mushroom preservation and flavor.

## 1 Introduction

**Fig. 1:**
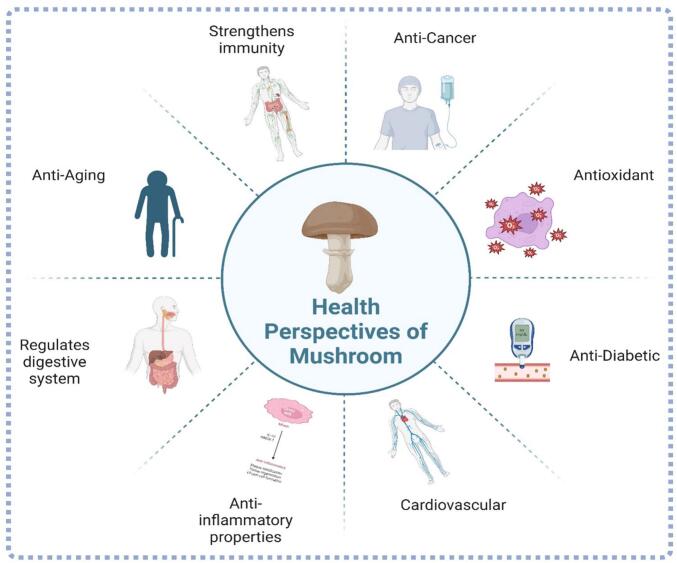
Major health benefits of Mushrooms.

Mushroom cultivation presents a profitable agribusiness opportunity that addresses several key issues, including resource utilization, circular economy, job creation, sustainability, and nutritional security, while also mitigating the impacts of unpredictable weather (Bijla & Sharma, 2023). From a geographical perspective, mushrooms have been present on Earth since before the rise of humanity, as evidenced by fossil records from the late Cretaceous period (Feng et al., 2012). Asia is the leading region in global mushroom production, with China being the dominant producer. China accounts for approximately 80 % of the world's mushroom production (Li & Xu, 2022). In addition to China, countries like Japan and South Korea are also significant producers, with a focus on shiitake and enoki mushrooms. The United States and Canada are major players in the North American mushroom industry. The U.S. is known for its large-scale production of white button mushrooms, which dominate the market due to their versatility and consumer preference (Singh et al., 2020). Canada also has a significant mushroom industry, with production focused on white button, oyster, and shiitake mushrooms. European production is diverse, including white button, oyster, and specialty mushrooms like shiitake and portobello (Zalewska et al., 2018a). South American countries like Brazil and Argentina are emerging players in the global mushroom market. These countries are developing their mushroom industries with a focus on expanding production capacity and improving quality. The region is increasingly adopting modern cultivation techniques and exploring market opportunities for both local consumption and export (Thakur, 2020). Researchers have identified over 70,000 fungi species globally, with 2000 being edible, 10 % being poisonous, and a few being considered mortal (Yenealem et al., 2013). Mushrooms offer a range of nutritional and health benefits that contribute to their value as dietary supplements. In addition to being a good source of high-quality protein, mushrooms are rich in essential vitamins (such as B vitamins), minerals (including selenium, potassium, and copper), and bioactive compounds. These compounds include polysaccharides like beta-glucans, which have been associated with immune-boosting properties, and antioxidants that help combat oxidative stress. Furthermore, mushrooms contain unique phytonutrients with potential health benefits, such as anti-inflammatory, anti-cancer, and antimicrobial properties (Fig. 1). These diverse health-promoting attributes make mushrooms a valuable component of dietary supplements beyond just their protein content (Chang, 2006). Certain edible mushrooms offer protein levels that equal or exceed those found in animal sources like milk, eggs, meat, and fish, and are on par with the highest plant-based protein sources. Consequently, edible mushrooms represent a superior source of high-quality protein, which can be cultivated more efficiently, cost-effectively, and with a reduced environmental impact (González et al., 2020). The medicinal properties of mushrooms, including their antioxidant activity, ability to lower cholesterol, and potential to reduce the risk of cancers, high blood pressure, and hypercholesterolemia, enhance their utility for therapeutic purposes (Daba et al., 2008; Mekonen et al., 2015). Despite growing awareness of their nutritional and medicinal benefits and an increase in production (Chang, 2006), mushrooms have been used historically as food, medicine, and even as an intoxicant (Subramanian, 1995). However, the commercialization of mushrooms remains challenging due to their highly perishable nature, which includes their tenderness, high deterioration rate, and the need for immediate and proper processing after harvesting (Reddy, 2015). Because of these factors, mushrooms are often not stored or transported for more than 24 h in many parts of the year and in various regions (Rai & Arumuganathan, 2008). To sustain the growing mushroom farming and industry, appropriate postharvest methods for storage and processing are essential (Siddiq et al., 2018). Common preservation methods, such as drying, pickling, freezing, canning, and sterilization, are widely applied to extend the shelf life of mushrooms and their products (Biswas, 2022). Various innovative techniques have been developed to preserve freshness and enhance flavor, marking significant advancements in the mushroom industry (Rai & Arumuganathan, 2008). Effective methods such as High-Pressure Processing (HPP), freeze-drying, nuclear magnetic resonance (NMR), imaging technology, spectroscopy, and advanced packaging technologies have been introduced (Verma et al., 2022). By implementing suitable postharvest processing and preservation methods, it is possible to maintain the sensory characteristics and nutritional value of mushrooms effectively (Dawadi et al., 2022; Kumar et al., 2021). The mushroom processing industry is undergoing a revolution due to automation, which streamlines labor-intensive operations. Numerous studies have explored the advancement of innovative methods and their impact on mushroom quality. However, a comprehensive analysis of these innovative techniques—such as innovative packaging, electron beam radiation, edible coatings, ozone treatment, and other processing technologies—remains absent from the existing literature (Barbosa et al., 2020) and requires thorough review (Marçal et al., 2021). In light of this, the present review discusses various aspects of mushroom processing, innovative techniques, and their role in revolutionizing the mushroom industry. This review aims to provide insights that could enhance the commercialization of mushrooms both domestically and in international markets.

### 1.1 Significance of edible mushroom in the global food industry

Throughout ancient history, mushrooms have been pursued by humans as a food source (Cooke, 1977). Initially, their use was primarily for their palatable taste and unique flavors (Rai & Arumuganathan, 2008). However, contemporary consumption patterns have shifted due to increased awareness of their high nutritional and medicinal properties (Wasser, 2010). Various global studies have investigated the benefits of different types of mushrooms in combating various illnesses, as well as their historical uses (Abou-Samra et al., 1987; Rolfe & Rolfe, 1925; Houghton, 1995; Bano and Rajarathnam et al., 1998). The demand for mushroom products is growing significantly, driving business expansion. Globally, mushroom production exceeds ten billion tons, with Asia being the primary producer (76 %) (Veljović & Krstić, 2020). The worldwide production of mushrooms is increasing at an annual growth rate of 8 % (Singh et al., 2020). India, despite being a late entrant, has also seen rapid growth in mushroom production, exceeding one lakh tons, with an annual growth rate of 15 % or more (Bhumarkar, Mahajan, & Kumar, 2021). India exports approximately 25 % of the U.S. mushroom imports and has domesticated over 20 types of mushrooms (both edible and medicinal). Technological advancements have enhanced their commercial viability (Sharma et al., 2017), significantly boosting the global industrial importance of mushrooms.

Mushrooms contain substantial amounts of dietary fiber composed of diverse complex polysaccharides with prebiotic properties, highlighting their potential in both pharmaceutical and food sectors (Petrović et al., 2022). Edible mushrooms can be utilized in processed foodstuffs, either directly as an ingredient or indirectly as a fermentation source (Moon & Lo, 2014). They serve as natural alternatives to synthetic additives and enhancers in food products. Interest in processed mushroom products, including dried, fermented, and preserved/canned options, is increasing globally (Petrović, Kostić, Stojković and Glamočlija, 2022). Edible mushrooms have demonstrated the capacity to boost immunity and provide a nutritious diet (Jaloot, Owaid, Naeem, & Muslim, 2020), making mushroom processing highly relevant to the global food industry. In 2013, the global demand for natural supplements and nutraceuticals reached 176.7 billion USD, with mushroom-based products comprising 10 % of this market (Hoti et al., 2022). Consumer interest in mushroom-based products significantly influences their market at both national and international levels (Veljović & Krstić, 2020). The direct and indirect uses of edible mushrooms in various food products, as studied by various researchers, are detailed in Table 1. The growth and popularity of mushrooms are shifting from Asia to Western regions due to increased consumer preferences and demand (Tagbata & Sirieix, 2008). Research into consumer attitudes and factors that drive acceptance of mushroom-based products is crucial for enhancing market demand (Veljović & Krstić, 2020; Hoti et al., 2022). The increasing use of mushrooms and their bioactive components in both the food and pharmaceutical industries underscores the need for further research to understand usage determinants and boost global market reception. The growing awareness of the health benefits of mushrooms has led to increased demand, fostering market expansion. Innovations in cultivation techniques and the development of new mushroom varieties contribute to market growth. Overall, the significance of edible mushrooms in the global food industry is multifaceted, encompassing nutritional value, economic impact, culinary diversity, and sustainability. As consumer preferences shift towards healthier and more sustainable food options, the role of mushrooms is likely to continue expanding, contributing to both dietary and environmental improvements.

**Table 1:** Direct/Indirect use of edible mushrooms.

**Direct/Indirect use**	**Application**	**Variety**	**Characteristics**	**References**
Direct use	Bakery (Bread)	*Lentinus tuberregium*	Increased loaf volume	Lee et al., (2004)
	Functional bread; herbal tea, powder, dietary supplement	*Ganoderma lucidum*	Nutraceutical and Beneficial health effects	Ritota & Manzi., (2023); Ayuso et al. (2022), El Sheikha, 2022
	Fermented product	Grifola frondose	Accelerated alcohol fermentation by yeast	Chung et al. (2004)
	Bakery products (bread, cookies, Maize flour)	Fresh mushroom/powder of P. *ostreatus*; P. *pulmonarius*; *P. eryngii*; P. *sajor-caju* at different concentration	Improved functional properties and beneficial health effects	Kim et al., 2010, Okamura-Matsui, Tomoda, Fukuda and Ohsugi, 2003
	Papad/herbal seasoning/potato pudding/noodles	Powder of P. *sajor-caju/* P. *ostreatus*	beneficial health effects/effect functional properties	Ritota & Manzi, (2023).
Direct use	Meat based products	*Pleurotus spp* Fresh mushrooms (powder/flour/puree/paste/whole)	Effect nutritional/textural and functional properties	Ritota & Manzi, (2023).
Indirect	Bakery (cookies/Steamed buns)	Polysaccharide extract (*P. sapidus*)	Effect functional properties	Ritota & Manzi, (2023).
	Pasta	β-glucan-rich fraction (*P. eryngii*)	Decreased functional properties/Increased color value	
Direct /indirect	Ingredient in dairy products (powder and extract)	*Pleurotus spp*	Good prebiotic source	Ritota & Manzi,. (2023).
Indirect	Aqueous extract in low-fat yogurt	*P. ostreatus*	Improved functional and rheological properties	Ritota & Manzi,. (2023).
	β-glucans extract in Fat white brined cheese and Ovine soft spreadable cheese	*P. ostreatus*	Improved storage properties / Increased flavor during storage	
	Tocopherol -rich extract in yogurt	*P. ostreatus & P. eryngii*	Improved nutritional and functional properties	
Indirect Use	Fermentation (Cheese-like food)	*Schizophyllum commune*	Lactate dehydrogenase; milk clotting activity	Okamura-Matsui et al. (2001); Atiqur, et al.,(2023)
	(Soy milk)	*G. lucidum*	Used in soy milk fermentation	Yang and Zhang (2009); Kuppamuthu, et al., (2022)
Indirect Use	Processed fish meat	*P. cornucopiae,* *P. eryngii* (ergothioneine extract)	Anti-discoloration effect	Ritota & Manzi (2023).
Indirect Use	Additive (Apple juice) (Processed fish meat)	*Flammulina velutipes*; (enokitake) *F. velutipes; L. edodes*	Inhibition of browning in apple juice Stabilized color	Bao, Osako and Ohshima, 2010, Jang, Sanada, Ushio, Tanaka and Ohshima, 2002

According to FAOSTAT data from 2018, global production of mushroom-based products is steadily increasing, which is driving greater commercial growth in the mushroom industry. The total global production is estimated to exceed ten billion tons, with Asia being the dominant producer, accounting for nearly 76 %. The growing use of mushrooms in the food and pharmaceutical industries underscores the need for research into the factors influencing the utilization of mushroom-based products to enhance their global adoption.

Global sustainability presents numerous opportunities for innovative responses and product development. The current market faces a shortage of vegan protein sources, exacerbating the issue. To address this gap, microbial cultures must be more widely recognized. Microorganisms such as mycelium fungi and microalgae exhibit high protein content in their biomass. Given the presence of essential amino acids, these protein sources are reliable options for vegans. Mushroom proteins offer a favorable protein-to-calorie ratio, providing additional nutritional benefits. Furthermore, incorporating mushrooms into our diet can help reduce meat consumption and lower carbon emissions (Ahlborn et al., 2019).

## 2 Traditional processing techniques

Traditional processing methods such as drying, pickling, freezing, sterilization, and canning can significantly extend the shelf life of mushroom products and enhance their value. These techniques help mitigate price fluctuations during peak and off-seasons (Rai & Arumuganathan, 2008). Various conventional methods have been employed historically to preserve mushrooms, including blanching, shade drying, solar drying, hot air drying, canning, and pickling (Jayaraman & Gupta, 2020). Table 2 presents the effects of different processing techniques on the nutritional composition of mushrooms. Blanching is a pretreatment method applied before various food processing techniques such as drying, frying, freezing, and canning (Arroqui et al., 2003) and is essential in the processing of fruits and vegetables (Negi & Kumar Roy, 2001). Drying is an ancient method used to preserve food commodities for extended periods. Indigenous communities in Mexico have historically employed two primary methods for preserving wild mushrooms: drying and, to a lesser extent, pickling (Pérez-Ovando, 2017). Effective traditional processing techniques can reduce postharvest losses and enhance economic returns for growers and processors (Rai & Arumuganathan, 2008). Hot air drying is a prevalent technique in the edible mushroom industry due to its simplicity and cost-effectiveness, addressing the challenges associated with complex operations and minimal investment (Guo et al., 2021). Among the various preservation methods, canning is the most commonly used technique for commercial mushroom preservation (Rawson et al., 2011).

**Table 2:** Effects on the nutritional composition of mushrooms by different processing methods.

**Mushrooms type**	**Processing/Storage Method**	**Impact on Nutrition content**	**References**
*Agaricusbisporus*	Blanching (95–100 °C/15 min.)	Decreased levels of minerals	Coskuner & Ozdemir, 2000
	Storage (12 °C/12 days)	Decreased sugar content, fructose, and mannitol; increased free amino acids (77.92–140.57 g/kg)	Tseng & Mau, 1999
*Macrolepiota procera*	Drying	Higher DPPH scavenging activity	Fernandes et al., 2013
*P. ostreatus*	Blanching (88 °C/1 min, Brining (25 % salt solution/ 30–60 min)	Reduction in protein, fat, and carbohydrate contents	Muyanja et al., 2014
*Macrolepiota mastoidea, Lactariusdeliciosus,* *Sarcodonimbricatus*, and *Macrolepiota procera*	Drying, freezing, and cooking	Improved antioxidant activities and nutrient concentrations in dried or frozen mushroom samples as compared to of cooked samples	Barros et al., 2007
*Amanita zambiana*	Frying, microwave heating, boiling, drying	• Frying increased proteins, lipids, and carbohydrates • Microwave heating increased the proteins and carbohydrates content Boiling increased the carbohydrate content and decreased the phenolic contents, • Drying increased the proteins, carbohydrates, and total phenolic components	Reid et al., 2017

### 2.1 Drying methods for preservation

Drying is a traditional and widely preferred method for preserving mushrooms (Walde et al., 2006; Wang et al., 2014). This process reduces moisture content, thereby inhibiting microbial activity and protecting against microbial contamination. Mushrooms are typically dehydrated until the moisture content falls below 10–12 % at a temperature of 55–60 °C to extend their shelf life (Yapar et al., 1990). Dehydrated mushrooms and mushroom powder are used in various food products, including premixes for instant soups, casseroles, pasta, snack seasonings, meat products, and rice dishes (Das et al., 2021). Srivastava et al. (2009) developed a blend of dried oyster mushroom powder (20 %) with corn flour (40 %), milk powder (25 %), salt (8 %), sugar (3 %), and black pepper and oregano (2 % each), resulting in a soup high in protein, minerals, and fiber, while being low in fat, carbohydrates, and energy value. Lu et al. (2018) integrated mushroom powder into pasta, enhancing its nutritional profile by increasing protein content, as well as soluble and insoluble dietary fiber, compared to durum wheat semolina. The inclusion of mushroom powder also significantly reduced starch breakdown. Dehydrating Pleurotus ostreatus fruiting bodies at 40 °C yields optimal rehydration properties (Apati et al., 2010).

Shade drying, which utilizes solar energy for heating, is another drying method (Deng et al., 2021). Similar to sun drying, shade drying involves placing the product in a shaded, well-ventilated area with low humidity and no direct sunlight (Nurhaslina et al., 2022). Despite the challenges associated with solar drying, it remains an inexpensive dehydration method (Tiwari, 2016). The drying of various mushrooms, including oyster, portobello, and red cracking bolete, has been studied (Kic, 2018). Mechanical drying has been introduced to overcome the limitations of natural drying methods like solar or shade drying, which can lead to darker and moisture-rich products. Oven drying at 55–60 °C to a moisture content of approximately 7–8 % has proven effective in minimizing mushroom spoilage during storage (Rai et al., 2005). Mechanical and industrial drying methods have been shown to improve the quality of dried mushrooms (Das & Arora, 2018). The physical, chemical, and bioactive properties of dried Boletus edulis are affected by drying temperature; higher temperatures negatively impact physical and chemical properties, while polysaccharides, polyphenols, and total flavonoids remain unaffected by varying drying temperatures (50, 60, and 70 °C) (Guo et al., 2021). The drying kinetics of Calocybe indica (milky mushroom) were studied at air temperatures of 50 °C, 55 °C, and 60 °C, with moisture content reduced to 11.34 %–13.13 % (w.b.). Higher temperatures were found to decrease drying times and lower activation energy (Arumuganathan et al., 2004). Various drying methods, including hot air, combined hot air, microwave vacuum, and freeze-drying, have been evaluated for their impact on mushroom product quality. Pre-treatment with potassium metabisulfite (0.25 %) and citric acid (0.1 %) for 5 min was effective in preventing browning during the drying process (Arumuganathan et al., 2004). Giri and Prasad (2007) compared the drying rate and rehydration properties of microwave-vacuum drying with conventional methods at different air temperatures (50 °C, 60 °C, and 70 °C). Their findings indicated that microwave-vacuum drying reduced drying time by 70–90 % and produced dried products with better rehydration qualities compared to convective air drying. Innovative technologies such as ultrasound, pulsed electric fields, and high pressure can enhance drying efficiency, improve quality, and offer environmental and economic benefits (Radojčin et al., 2021).

### 2.2 Canning

Canning is by far the most widely used method for preserving mushrooms (Deák & Farkas, 2013). The canning process is conducted according to standard commercial practices as outlined by Rawson et al. (2011). The antioxidant activity, soluble protein content, and pH of canned mushrooms are influenced by factors such as sterility, processing temperature, and canning duration. Higher retort temperatures (130 °C) have been found to reduce soluble protein levels, antioxidant activity, and pH values (Sharma et al., 2017). White button mushrooms are canned in various forms, including whole, halved, sliced, stemmed, and in pieces, depending on market demand, and are preferred for canning over other mushroom varieties (Rai & Arumuganathan, 2008). Lin et al. (2001) studied the effects of different pretreatments on the yield and quality of canned mushrooms, finding that soaking mushrooms in water prior to canning reduced losses and increased product yield. Additives such as ascorbic acid, EDTA, sulfur dioxide, and citric acid are useful for enhancing color during the canning process (Arumuganathan et al., 2004). Soaking and the use of chemical additives affect the yield and quality of canned mushrooms, resulting in satisfactory drained weight and improved color (Srivastava et al., 2009). The traditional canning method, which involves energy and water-intensive steps such as vacuum hydration, blanching (with water reuse), and sterilization, has been examined by Paudel et al. (2017). Their study revealed significant savings in heat input (28 %) and water usage (25 %), with reusing blanching water improving preservation and canning process yield by 9 %, while also conserving water and energy.

### 2.3 Pickling

Preserving mushrooms through pickling with spices, brine solution, and vinegar is a straightforward method that helps maintain their nutritional value and extend shelf life. Additionally, combining drying with pickling can effectively prolong shelf life while providing a meat substitute for soups and stews (Tanimola et al., 2022). The pickling process involves washing, slicing, and blanching mushrooms with 3 % salt water before transferring them to jars or bottles with brine, vinegar, sugar, and spices. Steaming for 60 min is also an effective method for mushroom preservation (Kakraliya & Choskit, 2021). Pretreatment with ascorbate solution before pickling results in optimal chemical composition and sensory properties (Temesgen & Workneh, 2015). Devi and Sunita (2020) studied the sensory attributes of mushroom pickles, including color, aroma, taste, texture, and acceptability, and found that vinegar-preserved pickles could be stored successfully for 30 days at ambient temperature with no microbial growth. Mushroom pickles have beneficial probiotic properties, serve as a good appetizer, and are suitable for all age groups (Devi & Sunita, 2020). The nutritional composition and sensory attributes of oven-dried versus pickled mushrooms differ significantly. Pickled mushrooms have higher moisture, fiber, fat, protein, and ash content compared to oven-dried mushrooms, which have lower carbohydrates and dry matter (Temesgen & Workneh, 2015). Ascorbate pretreatment increases protein, ash, and fat content, while osmotic solutions demonstrate the highest rehydration capacity. Combining ascorbate treatments improves mushroom quality compared to oven drying and osmotic pretreatments (Temesgen & Workneh, 2015).

### 2.4 Challenges and limitations of traditional methods

Mushrooms have a high perishability rate, leading to various post-harvest physiological and morphological changes that can render them unsuitable for consumption (Kic, 2018). Studies have explored the drying characteristics of oyster mushrooms (Pleurotus ostreatus) using both sun-drying and tunnel drying methods (Liu et al., 2022). The deterioration of mushroom quality is a multifaceted process influenced by both internal factors inherent to the mushrooms and external factors related to storage conditions. To maintain postharvest quality, it is highly recommended to employ hybrid approaches that integrate thermal techniques with physical or chemical methods, as well as innovative non-thermal technologies such as plasma, ultrasound, and high-pressure treatments in combination with traditional methods (Zhang et al., 2019).

## 3 Emerging innovative processing methods

Innovation significantly impacts productivity (Polder et al., 2009). Several machines are now available for sorting, feeding, stem-cutting, and sizing mushrooms. Automated mushroom trimming machines can produce mushroom cap slices with varying diameters and thicknesses (Suraweera et al., 2023). Contemporary mushroom processing equipment features integrated mechanisms for automatic feeding, root removal, and sorting based on cap diameter. Given their short shelf life, mushrooms require suitable methods for post-harvest processing and preservation. Traditional processing methods have various limitations; however, numerous innovative techniques have been developed for mushroom processing and preservation (Huo et al., 2023). These include high-pressure processing, electrofluidic drying, cold plasma treatment, as well as advanced packaging and coating technologies. Additionally, new quality detection techniques such as spectroscopy, imaging technology, and nuclear magnetic resonance (NMR) have been developed, offering rapid and effective processing and detection methods (Huo et al., 2023). Appropriate post-harvest processing and preservation techniques are crucial for maintaining the organoleptic and nutritional quality of mushrooms, as discussed below.

### 3.1 High-pressure processing (HPP) for mushroom processing

High-pressure processing (HPP) is a physical treatment that enhances the safety and longevity of plant-based foods by deactivating enzymes and microorganisms while preserving bioactive compounds with minimal impact on their nutritional and sensory qualities. Like other plant-based foods, physical modification of mushrooms is necessary to extend their applications. This involves the synergistic use of various physical methods to transform raw mushrooms into consumer-ready products. HPP is a notable physical processing method with significant potential in the food industry. It can maintain the quality of fresh foods, such as mushrooms, with minimal effects on flavor and nutrition (Norton & Sun, 2008). Currently, HPP is successfully applied to a range of products, including fruit juices, sauces, desserts, rice dishes, oysters, and meat products (Barba et al., 2017). Studies on the antioxidant activities of HPP-treated powders of *Agaricus chaxingu* have demonstrated increased free radical scavenging activity, chelating activity, and total antioxidant activity (Lv et al., 2014). HPP-treated mushroom powder exhibits lower viscosity, greater fluidity, and improved solubility of proteins and polysaccharides, making it suitable for use in convenience food products and food additives (Lv et al., 2014). Enzyme activity can be enhanced with pressure treatments at 600 MPa, while complete inactivation may require pressures as high as 950 MPa (Podolak et al., 2020). The impact of high pressure on the texture, color, and yield of mushrooms has been assessed. Mushrooms vacuumed prior to pressure treatment have better color values compared to HPP-treated mushrooms alone, and are similar to conventionally blanched mushrooms. Pressure-treated mushrooms also exhibit better firmness compared to blanched mushrooms, with similar yield (Matser et al., 2000). Table 3 presents a comparative analysis of traditional and innovative processing techniques for mushrooms.

**Table 3:** Comparative analysis of traditional and innovative processing techniques for mushrooms.

**Aspect**	**Traditional Processing Techniques**	**Innovative Processing Techniques**
**Method**	**Drying**: Air-drying, sun-drying, or oven-drying	**Freeze-Drying**: Sublimation process under vacuum.
	**Canning**: Preserving mushrooms in jars with heat treatment.	**High-Pressure Processing (HPP)**: Using high pressure to kill pathogens.
	**Pickling**: Preserving mushrooms in acidic solutions.	**Microwave-Assisted Extraction (MAE)**: Using microwave energy to extract bioactives
	**Fermentation**: Using microorganisms to ferment mushrooms.	**Supercritical Fluid Extraction (SFE)**: Using supercritical CO2 for extraction.
	**Blanching**: Briefly boiling mushrooms before freezing or canning.	**Ultrasound-Assisted Extraction (UAE)**: Using ultrasound waves to enhance extraction.
**Advantages**	**Drying**: Simple, cost-effective, extends shelf life.	**Freeze-Drying**: Retains most nutrients, flavor, and texture.
	**Canning**: Long shelf life, ready-to-use.	**HPP**: Preserves flavor and nutrients, extends shelf life.
	**Pickling**: Adds unique flavor, extends shelf life	**MAE**: Faster extraction, higher yields
	**Fermentation**: Enhances nutritional profile, develops unique flavors.	**SFE**: Extracts pure compounds, minimal solvent use.
	**Blanching**: Reduces microbial load, preserves color and texture.	**UAE**: Improves efficiency, reduces processing time.
**Limitations**	**Drying**: Potential loss of some nutrients, time-consuming.	**Freeze-Drying**: High cost, complex equipment.
	**Canning**: Loss of some heat-sensitive nutrients, changes in texture.	**HPP**: Expensive equipment, limited availability.
	**Pickling**: High sodium content, potential alteration in flavor.	**MAE**: Requires specific equipment and conditions.
	**Fermentation**: Requires controlled conditions, may alter flavor.	**SFE**: High initial investment, requires expertise.
	**Blanching**: Can affect texture, flavor changes.	**UAE**: Equipment can be costly.
**Applications**	**Drying**: Snack foods, soups, and seasoning.	**Freeze-Drying**: High-quality ingredient for instant meals, snacks.
	**Canning**: Ready-to-eat products, convenience foods.	**HPP**: Ready-to-eat products with extended shelf life.
	**Pickling**: Specialty foods, gourmet products.	**MAE:** Nutraceuticals, functional foods
	**Fermentation**: Health foods, traditional cuisines.	**SFE**: Extracts for supplements, flavors, and fragrances.
	**Blanching**: Preparation for freezing, pre-cooked products.	**UAE**: Enhanced bioactive extraction.

### 3.2 Freeze- drying and its impact on mushroom quality

Freeze drying is an effective method for drying and preserving mushrooms and other perishable food commodities with minimal quality losses compared to other drying methods (Nadew et al., 2024). Freeze drying can be used alone or in combination with other processing methods to achieve optimal results. The physical quality of vacuum freeze-dried button mushrooms has been studied by Jagadish et al. (2009) and Liu et al. (2016). Optimal freeze-drying parameters can produce dried food with chemical characteristics that closely resemble those of the raw material (Ciurzynska & Lenart, 2011). Freeze-drying process parameters significantly affect the quality of dried mushrooms, including attributes such as protein content, ascorbic acid, and antioxidant activity (Tarafdar et al., 2017). During optimization studies using Response Surface Methodology (RSM), it was observed that freeze-drying could retain up to 86 % of the ascorbic acid in mushrooms (Giri & Prasad, 2007). The antioxidant activity of freeze-dried mushrooms is influenced by process parameters (Tarafdar et al., 2017). Conventional hot air drying results in higher density in mushrooms compared to samples dried using combined or freeze-drying methods (Ren et al., 2023). This indicates that freeze drying is superior, as hot air drying can cause significant shrinkage and collapse of cell walls.

### 3.3 Innovative heat treatments for improved texture and flavor

Thermal methods, such as drying and cooling, are commonly used strategies to delay the deterioration of mushroom quality by controlling storage temperature and water activity (Pei et al., 2024). The impact of heat pump dehumidifier drying (HPD) on the sensory characteristics of shiitake mushrooms was found to be superior compared to hot air drying and vacuum freeze drying. This improvement is attributed to the partial inhibition of enzymatic and Maillard reactions, leading to higher levels of volatile sulfides and comparable umami content (Liu et al., 2022). Among traditional drying methods—hot air, microwave, vacuum freeze, and infrared drying—hot air drying is the simplest and most cost-efficient method that enhances the unique flavor of shiitake mushrooms through enzymatic and Maillard reactions. However, it can also result in undesirable nutritional and visual effects in the final products. In contrast, vacuum freeze drying maintains the nutrients and original shape of various mushroom species, such as shiitake, Pleurotus eryngii, and Agaricus bisporus, producing high-quality dehydrated mushrooms (Luo et al., 2022; Xu et al., 2021). Heat pump dehumidifier drying has been identified as the optimal process for producing top-quality mushroom products. This technology is noted for its minimal energy consumption, suitability for heat-sensitive items, and eco-friendly characteristics (Patel & Kar, 2012). Additionally, heat pump technology allows for the automatic adjustment of temperature and relative humidity of the warm air.

## 4 Advanced packaging techniques

Advanced packaging technologies such as Modified Atmosphere Packaging (MAP), Active Packaging (ACP), Biodegradable Film Packaging (BFP), and Nanocomposite Packaging (NCP) have been studied extensively to address the quality decline of fresh mushrooms (Feng, Heran, et al., 2023). These methods have been found to be effective in preserving mushroom quality. Nanoemulsion-based packaging has also been applied to various food products, though research on its use for mushroom preservation is limited. The next phase in packaging innovation involves exploring intelligent packaging solutions for edible mushrooms. MAP is the most widely utilized method for preserving edible mushrooms, with active packaging, nano packaging, and biodegradable film packaging also being prominent (Feng, Xu, et al., 2023). The effects of different packaging techniques on enzyme activity, antimicrobial activity, antioxidant activity, and respiration rate have been studied by Shonte et al. (2024). Their research observed the impact of these packaging strategies on the texture, color, nutritional value, and shelf life of mushrooms, demonstrating the effectiveness of these methods in preserving mushroom quality. Edible coatings not only enhance barrier properties and overall quality but also offer biocompatibility and environmental friendliness (Amin-Chowdhury et al., 2021; Louis et al., 2021; Marçal et al., 2021). The application of edible coatings has been shown to boost antioxidant activity and preserve the firmness, color, and total polyphenols of Agaricus bisporus mushrooms (Shonte et al., 2024). Recent advancements in packaging technologies involve utilizing a combination of methods to integrate active ingredients or nanoparticles into a bio-polymeric matrix, leading to the development of functional edible coatings, biodegradable packaging, active packaging, and nanocomposite packaging specifically designed for preserving edible mushrooms (Shonte et al., 2024).

### 4.1 Modified atmospheric packaging (MAP) for extending shelf life

Several studies have been conducted to mitigate the deterioration of mushroom quality post-harvest, including the use of Modified Atmosphere Packaging (MAP) (Belay et al., 2016). One study investigated the shelf life of button mushrooms (Agaricus bisporus) using MAP under various conditions, including different film types, storage durations, and MAP categories. This study evaluated the effects on quality parameters such as weight loss, texture, pH, color, and veil opening. The research revealed that browning of mushrooms could be effectively prevented with packages containing 10 % and 20 % oxygen and a film thickness of 44 μm (Zalewska et al., 2018b). The optimal conditions for extending mushroom shelf life were found to be 20 % oxygen, 0 % carbon dioxide, 80 % nitrogen, and a 44 μm thick packaging film. The composition of the headspace gas in MAP significantly affects the quality of both whole and sliced mushrooms (Ban et al., 2014). The use of PE-2 films combined with coating treatments resulted in desirable quality characteristics for preserved mushrooms, with successful packaging combinations extending the shelf life to 7 days at 12 °C, meeting consumer satisfaction. The preservation of fresh oyster mushrooms was also improved by MAP in combination with chemical treatments (Xiao et al., 2011). Low-Density Polyethylene (LDPE) was observed to be a more suitable packaging material compared to Polyvinyl Chloride (PVC) and LDPE-PVC blends for MAP storage. The combination of MAP with chemical treatments—sorbitol (0.05 g/100 g), CaCl2 (1.0 g/100 g), and citric acid (3.0 g/100 g)—was found to have inhibitory effects on weight loss and cell permeability during mushroom storage. MAP conditions of 1.5 % O2 and 20 % CO2, combined with these chemical treatments, were suggested to be beneficial in preserving quality (including PPO activity, texture, and organoleptic properties) and extending the shelf life of oyster mushrooms to 4–6 days (Xiao et al., 2011).

### 4.2 Active packaging incorporating antimicrobial agents

Antimicrobial packaging represents a promising application of active food packaging technology (Chen & Brody, 2013). This technology extends the shelf life of food products by inhibiting bacterial growth and preventing spoilage. Active packaging incorporating zeolite (clinoptilolite) and acai extract has demonstrated effectiveness in preserving various aspects of mushrooms. Compared to traditional packaging methods, active packaging enhances the chemical properties of mushrooms by increasing antioxidant activity, reducing moisture loss, and slowing the browning process both externally and internally. Hanula et al. (2021) suggested that integrating acai extract and zeolite into packaging materials can protect mushrooms from spoilage for longer periods. Additionally, the use of active packaging not only reduces the need for food preservatives but also supports environmental conservation by minimizing waste production.

## 5 Value added Mushroom products

The nutritional, medicinal, and functional properties of mushrooms enhance their value and encourage the development of value-added products (Barros et al., 2007). Sliced and dried mushrooms can be processed into powders that serve as dietary fiber additives in various foods and as partial replacements for wheat flour in bakery items. Dehydrated mushroom slices are versatile and can be used in soups, cookies, nuggets, and snacks. Additionally, mushroom-infused products such as bread, cakes, oil-roasted mushrooms, and mushroom pâté offer enhanced nutritional benefits and can promote greater consumption among children and the elderly (Ravi & Siddiq, 2011). For mushroom growers, incorporating value-added products into their offerings is essential. Successful developments include high-quality oyster mushroom preserves (murraba), mushroom ketchup, mushroom candy, and mushroom chips (Kawatra, 2014). These products not only reduce post-harvest losses but also increase income by providing consumers with nutritious, low-fat, and protein-rich food options (Arumuganathan et al., 2004) Fig. 2 illustrates various products prepared from mushrooms.

**Fig. 2:**
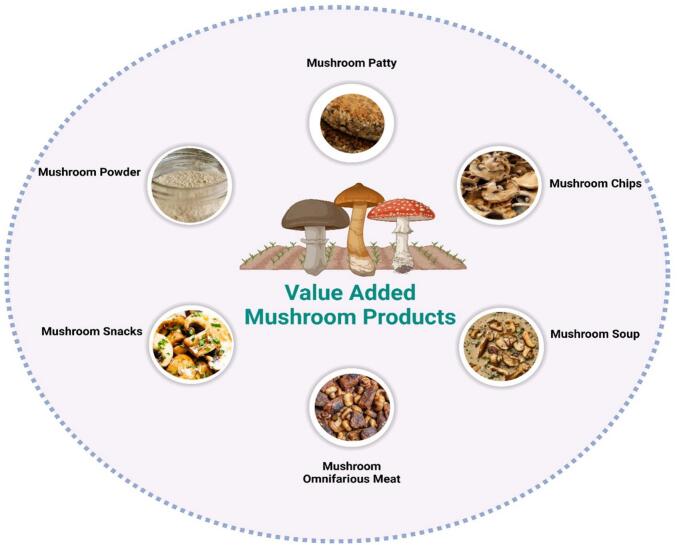
Various products prepared from mushrooms.

### 5.1 Mushroom powders

Mushroom powder indeed offers an exciting avenue for enhancing various food products. Its ability to boost protein content, dietary fiber, and minerals makes it a valuable ingredient for improving the nutritional profile of baked goods and other food products. The sensory attributes and quality of baked items can be significantly enhanced with the right formulation, as demonstrated by various studies. For example, incorporating 5 % powdered oyster mushroom into wheat bread can enhance texture and nutritional quality, while adding 4–10 % mushroom powder to sponge cakes has shown to yield favorable sensory attributes. Similarly, substituting wheat flour with mushroom powder and sweet potato flour in biscuits results in improved sensory characteristics and increased mineral content. These findings highlight the potential of mushroom powders to enrich food products not only nutritionally but also in terms of taste and texture.

#### 5.1.1 Challenges and limitations in the use of mushroom powder

**High Production Costs:** The production of mushroom powder involves several costly steps, including harvesting, cleaning, drying, and grinding. Specialized drying techniques, such as freeze-drying or hot-air drying, are often required to preserve the nutritional content and flavor of the mushrooms, but these processes can be expensive. Additionally, the initial investment in high-quality processing equipment and the ongoing costs of raw materials contribute to the overall expense.

**Scalability Issues:** Scaling up production from small batches to industrial quantities while maintaining consistent quality is challenging. Large-scale production can lead to variations in the powder's texture, flavor, and nutritional profile due to inconsistencies in raw material quality and processing conditions. Ensuring uniformity across large batches requires stringent quality control measures and potentially complex adjustments in processing techniques.

**Quality and Nutritional Stability:** Mushroom powder's nutritional value, including its vitamin and mineral content, can degrade over time due to factors such as exposure to light, moisture, and air. Ensuring the stability of these nutrients throughout the shelf life of the product requires careful packaging and storage solutions. Moreover, the sensory properties of the powder, such as flavor and aroma, may also diminish over time, affecting its appeal and effectiveness in food applications.

**Storage and Shelf Life:** Proper storage conditions are essential to maintain the quality of mushroom powder. High humidity and fluctuating temperatures can lead to clumping, spoilage, or loss of potency. Effective preservation methods must be employed to extend shelf life, which can add further costs and complexity to the production process.

**Market Acceptance:** Despite its benefits, mushroom powder may face challenges in market acceptance due to consumer perceptions and unfamiliarity with the product. Educating consumers about the benefits and applications of mushroom powder is necessary to enhance its marketability and adoption.

### 5.2 Mushroom-based snacks and convenience foods

The increasing demand for mushroom-based foods reflects their versatility and growing recognition of their nutritional benefits. The development of mushroom-based snacks, such as mushroom tikki, stuffed mushrooms, and mushroom chips, showcases their potential to offer both excellent sensory properties and nutritional advantages. These products are particularly valuable for their high content of protein, dietary fiber, antioxidants, and phenolic components, which are beneficial for maintaining a balanced diet, especially for children and adolescents. The innovation in creating mushroom-based flours, snacks, and imitation meat highlights a promising trend in food technology. Incorporating mushroom powder into various products like biscuits, laddoos, and candies can enhance their nutritional profiles while also extending their shelf life. As the market for value-added agricultural products grows, leveraging mushrooms for diverse applications can offer nutrient-rich options that cater to different consumer preferences and age groups. Overall, mushrooms' ability to be transformed into a range of value-added products aligns with the increasing interest in health-conscious and functional foods, making them a key ingredient in modern nutrition.

### 5.3 Utilization of Mushroom by products for sustainable product development

The management of mushroom by-products through conventional waste disposal methods has garnered considerable interest. These by-products have been utilized as raw or functional components in the food industry and in the production of livestock and poultry feed (Guo et al., 2022). Additionally, they have been employed in the manufacture of electrochemical materials, papermaking materials, ethanol, and other forms of bioenergy. Furthermore, these by-products have proven effective as absorbents in sewage treatment and as fertilizers for soil amendment. Mushroom processing by-products exhibit significant versatility and can be applied across a wide range of industries. To enhance productivity, it is advisable to explore innovative extraction methods, such as supercritical fluid extraction and microwave-assisted extraction, which can improve the bioactive substance levels in the by-products. Refining processing parameters, including temperature and duration, can also lead to higher quality end products. Spent mushroom substrate (SMS) is a complex mixture of organic material and mycelium that remains after mushroom cultivation. Laccase extracted from SMS plays a significant role in influencing the color of beverages by modulating the formation of phenolic compounds (Rajavat et al., 2020). Mushroom residues are utilized as by-products for extracting specific compounds for various purposes. This residue generally contains a substantial amount of water and nutrients, creating an environment conducive to the growth and rapid decomposition of microorganisms (Tian et al., 2017). In the food sector, mushroom by-products are primarily processed through cleansing, steaming, and grinding of misshapen mushrooms or mushroom stems, which are then used in a variety of food items (Guo et al., 2022). Currently, mushroom by-products are predominantly utilized in the production of bakery and meat products. Further research is needed to explore cost-effective utilization methods for these by-products and to evaluate their feasibility for industrial-scale applications.

## 6 Fermentation process

Mushroom fermentation offers potential benefits across various domains. Mushroom mycelia exhibit remarkable antioxidant activity and can enhance the levels of aglycons (daidzein, glycitein, and genistein) in fermented soybeans (Suruga et al., 2022). The fermentation process yields bioactive metabolites with medicinal and nutraceutical properties (Trovatti, 2013). Additionally, mushroom fermentation can be utilized to create a fully fermented bacterial cellulose membrane that incorporates mushroom fermentation extract, providing natural, safe, and multifunctional properties (Owaid et al., 2017). Furthermore, mushroom fermentation can be employed to produce bio-organic fertilizers using mushroom residue and straw. This method optimizes fermentation conditions for microorganisms, leading to reduced fermentation time and production costs (Suruga et al., 2022).

### 6.1 Fermented Mushroom products and their health benefits

Preserving mushrooms is crucial due to their desirable sensory qualities and the abundance of bioactive compounds with therapeutic and health-enhancing properties (Jabłońska-Ryś et al., 2019). Fermented mushrooms have been historically valued in many parts of the world and continue to be a sought-after delicacy (Jabłońska-Ryś et al., 2019). To preserve both wild and cultivated edible fungi, various conventional techniques have been adopted in recent times (Hua et al., 2017).

Lactic fermentation is a straightforward biotechnological method for preserving mushrooms while enhancing their nutritional value and functional characteristics (Ogidi & Agbaje, 2021). Lacto-fermented mushrooms are highly regarded in Southeast Asian countries, where fermentation techniques are commonly employed in food manufacturing to repurpose waste or inedible substances (Ortiz-Sanchez et al., 2023). For instance, milk caps of *Lactarius* are frequently used for fermentation, with certain species such as *L. rufus* and L. *torminosus* being toxic in their natural state and requiring fermentation for detoxification (Sõukand et al., 2015). Certain types of mushrooms can produce alcohol dehydrogenase, allowing for the creation of wine using mushrooms instead of *S. cerevisiae* (Okamura et al., 2001). Successful wine production with *A. blazei* (a mushroom variety) has been reported, with the wine containing approximately 0.68 % β-D-glucan, a compound known for its potential anti-cancer properties (Okamura et al., 2001). This study suggests that mushroom-based wine can be considered a functional food with potential cancer-preventative effects. Fermented sausages can be made from oyster mushrooms using traditional recipes for fermented pork sausage (Yang et al., 2001; Kim et al., 2009). However, the end product may exhibit bitterness due to the presence of certain amino acids, which can be reduced by soaking mushrooms in brine or vinegar (Arora, 1986; Rinaldi & Tyndalo, 1974). Incorporating fermented dairy permeate and mushroom powder into pan bread can enhance its quality and nutritional value, resulting in higher protein and mineral contents (Khider et al., 2015; Okafor et al., 2012). Traditional bread often lacks sufficient protein to meet human nutritional needs, making the addition of mushroom powder an effective strategy for boosting protein and nutrient levels in wheat bread (Agu et al., 2010; Okafor et al., 2012).

### 6.2 Challenges and opportunities in scaling up mushroom fermentation

Expanding mushroom fermentation operations presents both challenges and opportunities. Mushrooms serve as a significant nutritional resource with diverse applications in food, pharmaceuticals, and biotechnology. Fermentation, which involves the controlled growth of fungi under specific conditions, can enhance the nutritional profile of mushrooms and produce valuable secondary metabolites. During the fermentation process, yeasts and molds have been found to coexist with lactic acid bacteria (Liu et al., 2016). The concentration of yeasts in the fermented fungi typically peaks on either the fifth or sixth day of fermentation, or the fifteenth day for fungi pickled like kimchi at 4 °C. Yeasts can be a significant concern in food spoilage, particularly in environments with low pH, high salt levels, and low temperatures. Their presence during lactic fermentation can lead to an increase in pH, adversely affecting the overall quality of the food. Yeasts consume sugars that bacteria would otherwise ferment into lactic acid. Additionally, yeasts can use organic acids as a carbon source, resulting in decreased acidity and creating conditions conducive to the growth of spoilage-causing putrefactive bacteria (Franco & Perez-Dıaz, 2012; Satora & Celej). Despite these challenges, scaling up mushroom fermentation offers numerous opportunities for innovation, product diversification, and sustainable growth across various industries. Addressing these challenges through technological advancements, process optimization, and sustainable practices is essential to fully realizing the potential of large-scale mushroom fermentation.

## 7 Incorporation into other food products

Mushrooms are celebrated for their nutritional and medicinal benefits, contributing significantly to functional foods. Varieties such as button, oyster, shiitake, and enoki are popular globally, both as standalone items and as ingredients in various recipes (Dhanapal & Rajoo, 2023). They are commonly used to enhance the nutritional value of fortified foods like bread, noodles, pasta, biscuits, cookies, and soups. Additionally, mushrooms serve as valuable ingredients in chutneys, nuggets, jams, and jellies. The bioactive compounds in mushrooms are also utilized in traditional cosmetic products (Kumar, Bhardwaj, et al., 2022). Mushrooms can be incorporated into a variety of food products:

**Meat Alternatives**: Mushroom-based meat substitutes offer a healthier, plant-based option with a lower environmental impact compared to traditional meat products. Varieties such as portobello, oyster, and shiitake have a meat-like texture and savory taste, making them ideal for products like burgers, sausages, meatballs, and meatloaf (Brown, 2009).

**Soups and Broths**: Mushrooms add richness and depth of flavor to soups, broths, and stews. Dried mushrooms can be rehydrated and added to homemade or commercially prepared soups and broths to enhance their taste and nutritional profile (Bell, 2008).

**Pasta and Noodles**: Mushroom powders or finely chopped mushrooms can be incorporated into pasta dough or noodle recipes to create mushroom-infused pasta or noodles. Mushroom-filled ravioli or dumplings highlight the earthy flavor and versatility of mushrooms (Brodeur, 2005).

**Pizza Toppings**: Sliced or diced mushrooms are classic pizza toppings that add a savory umami flavor to the dish.

**Sauces and Condiments**: Mushrooms can be pureed or finely chopped and incorporated into sauces, gravies, and condiments to add richness, depth of flavor, and nutritional value. Mushroom-based sauces are suitable for pasta, rice, vegetables, or grilled meats (Bessette & Bessette, 1993).

**Bakery Products**: Finely ground mushroom powder can be used to enrich the nutritional value and taste of bread, muffins, and pastries. Mushroom powder can seamlessly blend into baked goods, enhancing them with vitamins, minerals, and antioxidants (Sławińska et al., 2022).

**Snack Foods**: Seasoned and roasted dried mushrooms can create savory snacks with a crispy texture and intense flavor. Mushroom chips, crisps, and trail mixes serve as wholesome, convenient snack options for consumers seeking alternatives (Jones & Jones, 2014).

**Dairy and Plant-Based Products**: Mushroom extracts or powders can be added to dairy items like cheese, yogurt, or cream-based sauces to improve taste and nutritional value. Additionally, mushroom-based ingredients can be used in plant-based dairy substitutes like almond milk, coconut yogurt, or cashew cheese.

**Beverages**: Mushrooms contain substantial water content, aiding hydration and promoting healthy digestion (Bellini et al., 2021; Kumar et al., 2021). Incorporating mushrooms into beverages offers a unique way to leverage their benefits. Adding ingredients like beetroot extract and pomegranate juice can enhance the flavor of mushroom-based beverages (Butu & Rodino, 2019). The growing demand for health-focused beverages is driving the development of innovative products that offer improved nutritional and functional properties. Mushroom extracts or powders can be used in smoothies, shakes, teas, and functional drinks to enhance flavor and nutritional content.

Utilizing mushrooms in diverse food items allows for creativity, differentiation, and meets consumer demands for healthy, flavorful, and eco-friendly foods. The adaptability, nutritional benefits, and unique taste of mushrooms make them a valuable component in enhancing the flavor and nutritional value of various food items. Despite the extensive use of mushrooms in products like chips, jams, patties, biscuits, powders, and dehydrated cubes (Parul Bora & Asha Kawatra, 2014), the exploration of mushroom-based beverages remains an area with significant potential.

Fusion products that combine mushrooms with traditional ingredients can offer a blend of nutrition, taste, and physiological benefits (Zhang et al., 2013). Mushrooms have a distinctive and enjoyable savory taste, known as umami, due to the presence of sodium salts of free amino acids like glutamic and aspartic acids, as well as 5′-nucleotides. This umami taste enhances the overall flavor of foods, making mushrooms a highly favored ingredient in a variety of culinary applications (Mau, 2005).

## 8 Technological integrations

Mushrooms have garnered significant attention not only for their culinary and medicinal uses but also for their potential in various technological applications (Zięba et al., 2020). Areas where mushrooms are being integrated include bioremediation, materials science, biotechnology, and food technology. In environmental applications, such as bioremediation, mushrooms have demonstrated the ability to clean up pollutants from soil and water (Harms et al., 2011). They can degrade and absorb a range of contaminants, including oil, heavy metals, and pesticides.

In materials science, mushrooms possess unique properties that make them suitable for producing strong, durable, biodegradable, and eco-friendly materials. Mycelium, the vegetative part of the fungus, can be cultivated into materials used for packaging, construction, insulation, and even as an alternative to leather (Mojumdar et al., 2021). In biotechnology, mushrooms hold potential for applications such as the production of enzymes, pharmaceuticals, and biofuels. They can be genetically engineered to produce specific compounds or to enhance their properties for various purposes (Llanaj et al., 2023). In the food industry, mushrooms are being utilized in innovative ways, including in plant-based meat substitutes, as flavor enhancers, and as a source of alternative protein, among other applications.

### 8.1 Utilization of AI in mushroom cultivation and processing

Mushroom farming and cultivation are becoming increasingly popular and sophisticated (Rahman et al., 2022). A crucial aspect of mushroom growth is weather monitoring and management, particularly with respect to humidity and temperature. In many rural areas worldwide, mushrooms are cultivated using traditional methods, which are often labor-intensive and susceptible to issues such as the growth of toxic mushrooms due to inadequate monitoring of weather and cultivation processes (Zhang et al., 2014).

To address these challenges, machine learning (ML) technology has been extensively utilized to classify edible mushrooms and prevent the proliferation of hazardous ones. In Bangladesh, for instance, IoT-enabled machine learning and automation are employed to classify poisonous mushrooms and manage mushroom farms. The Journal of Agriculture and Food Research (2024) proposes an IoT and ML-based irrigation system automation solution to address issues related to crop-specific irrigation, soil erosion, and over-irrigation. This solution employs a wireless sensor network with various sensor modules for monitoring and uses machine learning algorithms to predict the required amount of irrigation based on crop type and weather conditions.

### 8.2 Automation and robotics in mushroom harvesting and processing

Automation and robotics offer numerous benefits to the mushroom industry, including increased productivity, consistent quality, reduced labor costs, and enhanced food safety standards (Balan et al., 2022). With technological advancements, further innovations are anticipated to streamline mushroom production and meet the growing demand for this nutritious and versatile food source. Automation and robotics are increasingly utilized in various aspects of mushroom harvesting and processing, revolutionizing the industry in several ways: harvesting, sorting and grading, packaging, quality control, transportation, cleaning and sanitization, and data analytics and optimization. In mushroom harvesting for the fresh market, various consecutive tasks are either performed manually or by robots. Robotic picking involves identifying suitable targets based on size and location, picking the mushrooms without damaging or contaminating them or their neighbors, trimming the stipe, and placing them carefully into containers (Huang et al., 2021; Rowley, 2009). There is a significant need to develop automated methods for performing each of these tasks and to integrate these methods into a cohesive system. Smart technology for quality control and traceability in mushroom processing and cultivation has also been developed. This integrated network, facilitated by the Internet, connects generic objects into an accessible framework. The Internet of Things (IoT) provides a new platform that links computing devices, mechanical or digital machinery, animals, and people with unique identifiers (UIDs) for data exchange, minimizing the need for human-to-computer or human-to-human interaction (Kuzlu et al., 2021). The use of environmental control systems for edible fungi began with the adoption of such technology by Bells in 1947, setting a precedent for the environmental control of mushroom cultivation (Satyanarayana et al., 2019).

### 8.3 How technological integrations in the mushroom industry vary across different regions and markets?

Technological integrations in the mushroom industry vary significantly across different regions and markets, influenced by local economic conditions, infrastructure, and consumer preferences.

**Developed Markets:** In developed regions such as North America and Western Europe, technological advancements are extensively integrated into mushroom production and processing. Automation is prevalent, with advanced systems employed for harvesting, sorting, and packaging to increase efficiency and consistency (Sriram & Rajasekhar, 2022). High-pressure processing (HPP) and modified atmosphere packaging (MAP) are commonly used to extend shelf life and maintain product quality (Castellanos-Reyes et al., 2021). Developed markets also invest heavily in research and development, leading to innovations such as precision agriculture and sophisticated data analytics to optimize cultivation conditions (Iqbal et al., 2022). Additionally, sustainability is a major focus, with many producers adopting eco-friendly technologies and practices to minimize environmental impact (Zalewska et al., 2018b).

**Emerging Markets:** In emerging markets like China and India, technological integration is evolving. Traditional cultivation methods are still widely used, but there is a growing adoption of modern technologies such as improved substrate materials and controlled-environment agriculture to boost yields (Jayaraman et al., 2024). Although advanced technologies like automation and HPP are less common due to cost constraints, there is increasing interest in these areas. Local adaptations and innovations are being developed to address specific regional challenges, such as optimizing mushroom growth in diverse agro-climatic conditions (Qu & Jin, 2022). The adoption of these technologies is often supported by government initiatives and international collaborations.

**Developing Regions:** In less developed regions, technological integration is often limited by resources and infrastructure. Small-scale and subsistence farming practices dominate, with basic cultivation methods prevailing. However, there is potential for growth through the introduction of low-cost innovations and improvements in processing techniques. Efforts by international organizations and NGOs are crucial in introducing efficient and sustainable practices, such as simple preservation methods and improved cultivation techniques (Starck et al., 2024). Local innovations that address specific regional challenges are vital for overcoming infrastructure and resource limitations.

**Regional Variations:** Within countries, regional differences also impact technological integration. For example, in the U.S., regions with high-tech agricultural hubs, such as California, see faster adoption of advanced technologies compared to other areas (Sriram & Rajasekhar, 2022). In Europe, regions with strong research institutions, like the Netherlands, lead in implementing cutting-edge technologies and sustainable practices (Zalewska et al., 2018a). Conversely, regions with limited technological resources may rely more on traditional methods and incremental improvements.

## 9 Sustainable practices in Mushroom processing

Sustainable methods in mushroom processing encompass a range of approaches aimed at reducing environmental impact, enhancing resource utilization, and promoting social responsibility (Banasik et al., 2017). A key aspect of these practices is the efficient use of byproducts and waste streams. Processing facilities can implement systems to recycle the substrate used in mushroom cultivation, composting it to produce valuable soil amendments or feedstock for other industries. Utilizing energy-efficient technologies and renewable energy sources to support mushroom processing activities has been suggested as a means to reduce carbon emissions (Farashah et al., 2013). Additionally, integrating water-saving methods and water recycling systems contributes to sustainability initiatives. Chamberlain et al. (2020) highlighted the importance of fair labor practices and community support in sustainable mushroom processing. By prioritizing these aspects, the mushroom industry can significantly contribute to a more environmentally friendly and socially responsible food production system. In the cultivation process, agricultural waste is fully recycled and can serve as fertilizer for farms. This practice not only has the potential to generate additional income and employment opportunities but also adds value to the process. As a result, mushroom cultivation is increasingly prominent in rural and semi-urban areas, improving the socioeconomic conditions of farmers, rural youth, and particularly rural women, regardless of their educational background (Dey et al., 2020). Recycling and reusing processing by-products for use in the food industry, medicine, and cosmetics has been proven to be a sustainable practice. Energy-efficient processing technologies are revolutionizing the mushroom industry by significantly reducing energy consumption and environmental impact. These technologies include the implementation of heat recovery systems that effectively capture and repurpose waste heat produced during various processes (Rukhiran, Sutanthavibul, Boonsong, & Netinant, 2023). Advanced ventilation and air circulation systems ensure optimal climate control while minimizing energy usage, and high-efficiency lighting, such as LED fixtures, illuminate facilities with reduced electricity consumption. Moreover, investments in energy-efficient machinery and equipment, such as refrigeration systems and automated controls, further enhance efficiency throughout the processing chain. By integrating these technologies, mushroom processing facilities can achieve substantial energy savings, lower operating costs, and contribute to a more sustainable future. The use of mycelium-based biodegradable packaging represents an innovative and eco-friendly solution in the food industry (Angelova et al., 2021). Mycelial masses possess inherent strength properties that can be harnessed to produce a wide range of cost-effective materials for packaging, construction, food, and clothing. This environmentally friendly substitute for plastics can be used to create items like leather and plant-based edible steaks. Mycelium packaging, also known as myco-materials or mushroom packaging, offers numerous advantages, including sustainability, biodegradability, lightweight yet strong characteristics, customization options, and a positive brand image (Pohan et al., 2023).

## 10 Consumers perception and market trends

In recent years, there have been notable shifts in consumer perception and market trends within the mushroom industry (Zhang et al., 2014). Historically, mushrooms were known primarily for their earthy taste and nutritional benefits and were mainly used as a culinary ingredient. However, with evolving consumer preferences and a greater emphasis on health and sustainability, mushrooms have emerged as a versatile and environmentally friendly food choice. With their meaty texture and rich umami flavor, mushrooms have become a popular alternative to animal products. This trend has led to the creation of innovative products derived from mushrooms, including plant-based meat alternatives, mushroom jerky, and snacks made from mushrooms (Banach et al., 2023). In addition to their nutritional and medicinal benefits, mushrooms are increasingly recognized for their sustainability credentials (Martens et al., 2015). As a low-impact crop requiring minimal resources for cultivation, mushrooms offer an environmentally friendly alternative to conventional protein sources. Specialty and exotic mushroom varieties, once considered niche products, are now becoming more mainstream as consumers seek unique culinary experiences and flavors. Overall, the perception of mushrooms has evolved from a conventional cooking ingredient to a versatile, healthy, and eco-friendly food choice. The growing demand for innovative mushroom products underscores the potential of mushrooms as a highly nutritious vegetable that can address contemporary food and nutritional security issues (Thakur, 2020).

### 10.1 Market opportunities and challenges for Mushroom processors

The increasing consumer demand for mushrooms and mushroom-based products presents significant opportunities. This demand is driven by factors such as the rising popularity of plant-based diets, growing awareness of the health benefits associated with mushrooms, and their versatility in various culinary applications. This surge in demand opens up avenues for mushroom processors to diversify their product range, experiment with new recipes, and target emerging market sectors such as plant-based meat alternatives and functional foods. By implementing automation, quality control measures, and environmentally friendly packaging alternatives, mushroom processors can enhance operational efficiency, reduce costs, and strengthen their competitive position (Kim & Ruedy, 2023). However, mushroom processors face a combination of opportunities and challenges in the market (Rathod, 2023). Key challenges include:

**Perishable Nature**: Mushrooms are highly perishable, requiring careful handling and storage to maintain freshness and quality.

**Supply Chain Logistics**: Managing supply chain logistics and ensuring timely delivery to the market can be complex, particularly for fresh mushrooms.

**Raw Material Fluctuations**: Variability in raw material availability and pricing, along with seasonal variations in mushroom harvests, can impact operations (Mahawar et al., 2019).

**Market Competition**: Competition from both domestic and international suppliers intensifies pressure on processors to differentiate their products, maintain consistent quality, and offer competitive pricing (Shank & Govindarajan, 1993). By embracing innovation, adopting efficient processing technologies, and maintaining a focus on quality and sustainability, mushroom processors can position themselves for success in the dynamic and evolving marketplace.

### 10.2 Regulatory considerations

Regulatory considerations are crucial in mushroom cultivation and processing to ensure product safety and maintain consumer trust (Lu et al., 2020). Regulations primarily focus on food safety, agricultural practices, and labeling requirements (Henson & Caswell, 1999). Mushrooms intended for human consumption must adhere to specific standards during cultivation, harvesting, and processing to minimize contamination risks and ensure high quality. Labeling regulations specify the information that must be provided to consumers, including the origin, ingredients, and potential allergens present in mushroom products (Giusti et al., 2022). Compliance with these regulations enables growers and distributors to demonstrate their commitment to producing safe, high-quality products while adhering to legal standards.

### 10.3 Compliance with food safety regulations

Ensuring consumer safety and preserving the integrity of the mushroom industry is of paramount importance by adhering to food safety regulations (Pardo et al., 2017). Mushroom cultivation and processing operations must strictly comply with regulations governing hygiene practices, pest control, and sanitation standards to minimize contamination risks. Effective measures include proper handwashing, maintaining cleanliness in facilities, and controlling temperatures during storage and transportation to prevent the growth of harmful bacteria and fungi (Marriott & Robertson, 1997). Additionally, growers must monitor water quality, use approved pesticides, and implement Hazard Analysis and Critical Control Points (HACCP) systems to identify and mitigate potential risks throughout the production process. By prioritizing compliance with food safety standards, mushroom producers can protect consumers from foodborne illnesses and uphold their reputation for providing safe and high-quality products in accordance with regulatory requirements.

### 10.4 Labeling and marketing of innovative mushroom products

Marketing and labeling of new mushroom products present both challenges and opportunities in the food industry. The growing demand for mushroom-based products, driven by consumers' increasing focus on health and sustainability, highlights the need for accurate and informative labeling. Labels should provide detailed information on ingredients, nutritional value, and any unique qualities of the mushroom-based item. Additionally, transparent labeling of sourcing practices, such as organic or locally grown mushrooms, can attract consumers interested in ethically produced foods. Emphasizing sustainable cultivation methods, including their low environmental impact and efficient resource usage, can further appeal to environmentally conscious consumers (Kim & Ruedy, 2023). Innovative mushroom products can benefit from creative marketing strategies that highlight their versatility and culinary potential. By emphasizing the distinct flavors, textures, and health benefits of mushrooms, these products can attract culinary enthusiasts eager to explore new ingredients. Utilizing social media platforms and collaborating with influencers can generate excitement and effectively reach target audiences. Partnerships with chefs, recipe creators, and food bloggers can showcase the diverse ways mushroom products can be integrated into various dishes, from plant-based options to gourmet cuisine (Zimberoff, 2021).

Additionally, employing storytelling as a marketing strategy can enhance the appeal of innovative mushroom products. Narrating the product's development process or highlighting the expertise of mushroom growers and the innovative production techniques involved can effectively convey a brand's mission, values, and commitment to quality and sustainability. This approach helps establish an emotional connection with consumers and differentiates the product in a competitive market.

### 10.5 Future regulatory trends in the mushroom processing industry

The mushroom processing industry is poised to encounter significant regulatory changes influenced by factors such as technological advancements, environmental concerns, and shifting consumer preferences (Rathod, 2023). Expected regulatory developments include a heightened focus on food safety standards, which will lead to stricter protocols aimed at mitigating contamination risks. Additionally, sustainability is likely to become a central theme, with regulations encouraging or mandating the adoption of eco-friendly practices throughout the mushroom production and processing cycle. This may involve initiatives to reduce waste, optimize resource utilization, and minimize environmental impact. Increased transparency in labeling will ensure that consumers have access to accurate information regarding ingredients, nutritional content, and sourcing practices (Freeman, 2015). Proactively embracing these regulatory changes will be crucial for industry stakeholders to ensure compliance, build trust, foster innovation, and meet the evolving demands of consumers and regulators.

### 10.6 Ongoing research in mushroom processing

Ongoing research in mushroom processing is advancing through innovative techniques and technologies aimed at improving efficiency and product quality. Key areas of focus include advanced preservation methods and automation. High-pressure processing (HPP) and modified atmosphere packaging (MAP) are being extensively studied for their ability to extend shelf life while preserving nutritional value and sensory properties. HPP, for instance, has been shown to effectively maintain the quality of mushrooms by minimizing microbial growth and enzymatic activity (Castellanos-Reyes et al., 2021; Iqbal et al., 2022). MAP, on the other hand, helps in maintaining freshness and extending the storage life by controlling the atmospheric composition around the mushrooms (Zalewska et al., 2018). Automation in mushroom processing is also a significant area of research, with advancements in robotics and automated systems designed to enhance scalability and consistency in harvesting, sorting, and packaging (Gómez et al., 2022). These technologies not only improve efficiency but also reduce labor costs and increase precision in processing. Moreover, technologies such as nuclear magnetic resonance (NMR) and spectroscopy are being utilized to better understand and maintain the chemical composition of mushrooms throughout processing. NMR and spectroscopy help in monitoring and preserving essential nutrients like vitamins and antioxidants, ensuring that the final product retains its nutritional quality (Qu & Jin, 2022; Zhang et al., 2023). Sustainability is also a key focus, with research exploring the use of agricultural waste and by-products for mushroom cultivation, which aligns with efforts to minimize environmental impact and enhance resource efficiency (Jayaraman et al., 2024). This integrated research approach aims to address the challenges in mushroom processing and contribute to a more sustainable and effective industry.

## 11 Conclusion

The strategic geographical locations around the world position various regions as key players in the mushroom industry, leveraging their unique advantages to excel in production and innovation. Mushrooms, known for being low in calories, sodium, fat, and cholesterol, are also rich in essential nutrients and bioactive compounds, making them a valuable component of a healthy diet. Their production is notably more sustainable compared to meat, with fewer environmental impacts. The mushroom industry benefits from robust support through research and development, government initiatives, and the efforts of policymakers and entrepreneurs. This support is reflected in the significant economic benefits of mushroom cultivation, which offers high yields and substantial returns, enhancing the livelihoods of farmers and contributing to the national economy. Recent increases in consumer preference for mushrooms and mushroom-based products have led to heightened demand and supply in India & abroad. This growing interest highlights the importance of mushrooms in addressing the current scarcity of plant-based protein sources. However, the perishable nature of mushrooms presents a challenge. Effective preservation methods, such as drying, freezing, canning, pickling, and sterilization, are crucial for extending shelf life. Emerging technologies like High Pressure Processing (HPP), Freeze-drying, and advanced packaging solutions like Modified Atmosphere Packaging (MAP) and Active Packaging with antimicrobial agents are revolutionizing the industry, maintaining freshness, and enhancing flavor. Furthermore, automation is transforming mushroom processing, streamlining labor-intensive tasks and increasing efficiency. As the industry continues to evolve, ongoing research and innovation will be key to addressing challenges and capitalizing on new opportunities. In conclusion, while the mushroom industry in India demonstrates impressive growth and potential, a focus on future advancements, global trends, and addressing key challenges will be essential for sustaining its success and expanding its impact both domestically and internationally.

## CRediT authorship contribution statement

**Sangeeta:** Writing – review & editing, Writing – original draft, Software, Methodology, Investigation, Formal analysis, Data curation, Conceptualization. **Dhriti Sharma:** Writing – review & editing, Writing – original draft, Visualization, Validation, Investigation, Data curation. **Seema Ramniwas:** Writing – review & editing, Writing – original draft, Validation, Formal analysis, Data curation. **Robert Mugabi:** Writing – review & editing, Writing – original draft, Visualization, Software, Methodology, Investigation, Conceptualization. **Jalal Uddin:** Conceptualization, Data curation, Funding acquisition, Software, Writing – review & editing. **Gulzar Ahmad Nayik:** Writing – review & editing, Writing – original draft, Supervision, Software, Methodology, Funding acquisition, Data curation, Conceptualization.

## Declaration of competing interest

The authors declare that they have no known competing financial interests or personal relationships that could have appeared to influence the work reported in this paper.

## Data Availability

All the authors declare that if more data is required, then the data will be provided on a request basis.
